# The influence of riparian woody vegetation on bankfull alluvial river morphodynamics

**DOI:** 10.1038/s41598-022-22846-1

**Published:** 2022-10-28

**Authors:** Gergely T. Török, Gary Parker

**Affiliations:** 1 ELKH-BME Water Management Research Group, Eötvös Lóránd Research Network, Budapest, Hungary; 2 Department of Civil and Environmental Engineering, Department of Geology, University of Illinois at Urbana-Champaign, Urbana, USA; 3 National Laboratory for Water Science and Water Security, Department of Hydraulic and Water Resources Engineering, Budapest University of Technology and Economics, Budapest, Hungary

**Keywords:** Environmental impact, Hydrology, Geomorphology, Sedimentology

## Abstract

Exploring the effects of bank vegetation on fluvial morphodynamics has long been an essential part of fluvial morphodynamic-related research. In a practical sense, a central question is: does increased vegetation density increase or decrease the channel width? Several aspects concerning the role of vegetation may result in examples of both width decrease and increase. In this study, we examined more than 170 alluvial river sections. Our goal was to detect the phenomena that ultimately determine riparian woody vegetation-induced width variation. We found that bed material is a governing factor. In the case of fine-grained material, i.e. median size *D*_50_ < 2 mm, increasingly densely forested riparian vegetation reduces the bankfull Shields number, and destabilizes the banks toward a wider bankfull channel. In the case of coarse-grained material (i.e. median size *D*_50_ ≥ 16 mm), the effect is the opposite; increased density is correlated with a higher bankfull Shields number and a narrower bankfull channel. The extent of the role of vegetation varies depending on the ratio of characteristic root zone depth to channel depth and channel width. We present an improved estimator for bankfull Shields number, which considers riparian vegetation density. The bankfull Shields number can be estimated up to 19% more accurately with our corrected estimator.

## Introduction

When exploring the relationship between vegetation, flow and morphodynamic processes in rivers, a central question pertains to how vegetation influences their hydraulic geometry, and in particular their width^1^. Site-specific field and laboratory studies^2–7^ show examples of both vegetation-induced river channel widening and narrowing. Multiple processes come into play when vegetation interacts with rivers^8^. For instance, in the case of submerged and emergent vegetation along the river bank and floodplain, vegetation can act as obstacles that potentially cause an increment in turbulence in a zone above and around the roots or trunks. This may decrease local riverbed stability^5,7^ and lead to a wider channel. Vegetation, however, interacts not only with the flow, but also directly with sediment. This interaction, in turn, can cause deposition, thus increasing the stability of the riverbed particles^9,10^ and leading to a narrower channel. Ultimately, the superposition of vegetally mediated processes acts in concert with water and sediment to establish channel bankfull width. Relevant factors include vegetation density and type, and the structure of the root system itself^11^. Here we use data analysis of bankfull characteristics to extract some form of behavioral simplicity from the complexity of river-sediment interaction.

The appearance of roots leads to additional components of shear arising between the root system and the soil in the case of river banks, in the same way as in the case of hillslopes. (The same stability model can be used in both cases)^4,12–15^. Several experiments on root pullout force indicate that the root system induces apparent cohesion in the soil, although the precisevalue of the added cohesion cannot be directly deduced from the pullout force^14,16,17^. The presence^16^ of the binding action of roots can significantly increase (or its absence can decrease^18^) the stability of hillslopes (or river banks), especially in granular, friction-dominated soil. This added apparent cohesion depends on bond failure between the roots and the soil^17^. The strength of root reinforcement (added cohesion) can typically fall in the order of tens of kPa. For example, Burroughs and Thomas report values between 7.5 and 17.5 kPa^19^, and Schmidt et al.^16^ present values between 21.1 and 27.0 kPa. These values were determined for granular, friction-dominated forested hillslopes, where root breakage is the typical failure mechanism. In fine-textured sediment including cohesive material, the pull-out resistance takes a significantly lower value. The model of Waldron and Dakessian^17^ and the riparian vegetation measurements of Yu et al.^20^ indicate that maximum root reinforcement typically falls below 6 kPa^17^, because root slippage is the typical mode of failure.

Riparian plants can increase bank stability, but in so far as their weight increases the load on the bank, they can also decrease bank stability. In old-growth forests, for example trees can weigh up to 150 kN^21^. Bank stability analyses have been performed based on these values^22,23^. These analyses suggest that woody vegetation may cause river bank stability to differ between granular and cohesive bank material. In the case of sufficiently coarse material, the presence of trees consistently results in overall stabilizing of the riverbanks such that the equilibrium channel width is lower than otherwise. This is because the root system delivers higher reinforcement than the loss of stability engendered by the weight of trees. However, in the case of cohesive material, trees can reduce stability^24^ because the weight of mature trees adds a higher additional load to the bank that counteracts root reinforcement. This state of stability further deteriorates when the cohesive soil becomes saturated at bankfull water stage, and thus may result in a wider channel than otherwise. A potential explanation for this phenomenon is that the soil shearing resistance^25^ and the roughness between the roots and the soil^17^ take minimum values in saturated cohesive soils^13^.

For instance, the field measurements of Abernethy and Rutherfurd^26^ indicate that in the case of a cohesive river bank, the presence of trees can contribute to instability. Although tree weight does not play the most significant role in stability, it can be decisive where the bank itself is inherently unstable^26^. The relevant instability mechanism may be characterized by e.g., the presence of tree trunks sliding from the shore into the bed^7,27^. In contrast, Krzeminska et al.^28^ argued that in the case of granular soil, trees contribute very significantly to stability despite their weight.

Studies on root-induced added cohesion have also shown that this added cohesion decreases with depth due to the limitation of of root zone depth itself, as well as the change in root density with increasing depth^15,29–31^. Stromberg^32^ compiled a large database of 125 plant species specifically in regard to riparian vegetation. Hydroriparian species were found to have the shortest root system, being at most a few 10 cm long. The median root depth of annual and herbaceous plants is 0.43 and 0.65 m, respectively. Compared to these, the median root depth of shrubs and trees is between 3 and 4 m^32^ (The data set of 107 deciduous and 40 evergreen tree species (not specifically riparian) showed an average tree root depth of 3.34 m^33^).

Shrubs and trees have longer root systems and more local weight per unit area than annual grasses and herbaceous vegetation (including hydroriparian specimens)^32^. When they are present, then, shrubs and trees may exert a strong effect on bank stability.

## Hypothesis and approach

This study is formulated based on the following hypotheses. (1) When the bed, and thus basal bank material is sufficiently coarse (so the bank material is granular and, friction-dominated), increasing near-bank floodplain forest cover (i.e. woody vegetation including trees and shrubs) stabilizes riverbanks, and therefore reduces equilibrium bankfull width. (2) When the bed, and thus basal bank material is sufficiently fine, however the stability of the riverbank somewhat decreases, and the equilibrium bankfull width is rendered wider as woody vegetation density increases.

We validate our hypothesis below based on reported morphodynamic data for a total of 175 alluvial river sections (“Methods”). The data set covers a wide range of morphodynamic characteristics, i.e., bed material, but does not usually include separate data on bank material. Therefore, we assume that the basal bank material correlates with the reported bed material. We studied only river sections where riparian vegetation can play a role in morphodynamic processes. For this reason, we included neither bedrock rivers^34^ nor streams in permafrost^35^. Furthermore, in our present study, we could not take into account the fact that there are different plant species in each river section. We have taken the approach that the presence and density of woody vegetation are the key factors in our study. The bank vegetation density was determined manually, corresponding to the frontal area cover that can be determined on the basis of satellite images (“Methods” section). The statistical processing of the data set serves to demonstrate our hypothesis. Moreover, it allows us to recommend a refinement of the method of Li et al.^36^ for estimating morphodynamic characteristics. Thus, the effect of woody vegetation on river morphodynamics that we demonstrate here takes a form that can be directly applied to the prediction of river hydraulic geometry.

## Approach for quantifying our hypothesis

Our approach is based on morphodynamic parameters in terms of which the bankfull characteristics of a graded (equilibrium) river geometry can be easily expressed. First, we introduce these variables.

River bankfull geometry is characterized by the variables: bankfull width $B_{bf}$, bankfull depth $H_{bf}$ and streamwise bed slope $S$. River sections are also characterized by physical properties such as median bed material grain size $D_{50}$, submerged specific gravity of bed material $R$ and water kinematic viscosity ν. Water mass balance, sediment mass balance, and momentum balance can be described with these variables, which can be used to express the bankfull water discharge $Q_{bf}$ and the bed material load $Q_{tbf}$ at bankfull flow, as well as the formative dimensionless bankfull bed shear stress (Shields number) $\tau_{bf}^{*}$, which is here estimated as follows using the normal flow assumption^37^:

1 $$\tau_{bf}^{*} = \frac{{H_{bf} S}}{{RD_{50} }}.$$

Li et al.^36^ and Czapiga et al.^38^ have related *τ*_bf_* to streamwise bed slope *S* and dimensionless characteristic bed material grain size D*, where

2 $$D^{*} = \frac{{\left( {Rg} \right)^{1/3} }}{{\nu^{2/3} }}D_{50} .$$

Here we show that that the density of woody vegetation on the floodplain, as measured in terms of frontal area density, also influences bankfull Shields number. We then in turn show that vegetation influences bankfull channel width via the bankfull Shields number.

## Effect of woody vegetation on the bankfull Shields number

Many papers have been published demonstrating the dependency of the bankfull Shields number on various morphodynamic parameters in alluvial streams^36–40^. The results are consistent in that there is a strong correlation between the Shields number, the bed slope, and the bed material grain size. Li et al^36^ analyzed a data set of 230 river sections (including the data we use). Each river section corresponds to an alluvial, non-bedrock and non-permafrost river.

**Figure 1:**
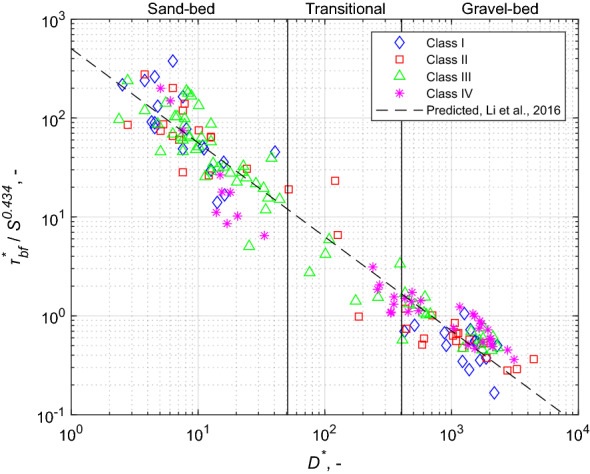
Regression relation of Li et al.^36^ (dashed gray line) and $\tau_{bf}^{*} , /S^{0.434}$ $D^{*}$, as well as data for the study river sections used here. The cases of the densest woody vegetation appears in purple (Class IV) and the tree- and shrub-free case is in blue (Class I), while the two intermediate states are in red (Class II) and green (Class III).

We were able to use only a subset of 175 reaches from the above data set, due limitations in locating some reaches and estimating vegetation cover for others (see “Methods” section). Figure 1 shows the ratio of the bankfull Shields number to slope as a function of the dimensionless grain size $D^{*}$ (Eq. 2) calculated from a data set of 175 river sections^41,42^. Also, the dashed gray line presents the Shields number predicted by Li et al.^36^, i.e.,

3 $$\tau_{bf}^{*} = 502D^{{{*} - 0.951}} S^{0.434} .$$

The exponents and coefficients in this equation were obtained from a combination of similarity and regression analysis^37^. Equation 3 demonstrates that the Shields number increases with slope (to about the half power) and decreases linearly with the bed material grain size.

Although the line follows the trend of the points, the scatter can be as significant as a half-order of magnitude. Regardless of the accuracy of the regression, additional factors need to be considered in order to understand the scatter. The point is underlined by the more recent work of Czapiga et al.^38^.

Hey and Thorne presented a classification scheme for floodplain vegetation based on the density (frontal area density) and type of bank vegetation^43^. On this basis, river sections can be divided into four vegetation categories according to density. We adopt the classification scheme of Hey and Thorne, as shown in Fig. 1. Details as to how we implemented this scheme are presented in the section “Methods” section. Riparian vegetation density was determined from aerial photographs by visual inspection of trees and shrubs along banklines (“Methods” section).

In terms of sediment size, we classified river sections based on Parker’s findings^44^. According to his examination of Canadian (Alberta)^45^ and Japanese rivers^46,47^, sand and gravel-dominated riverbeds separate from each other, and the following classification can be established: a bed with a median grain size of less than 2 mm can be classified as a fine-bedded river (sand-bed and silt-bed rivers), and a gravel-bed river with a grain size greater than 16 mm can be classified as coarse-bedded. Most river sections fall into these two classes. Between the two is a transient range (2 mm < $D_{50}$ < 16 mm) with relatively few river sections. We emphasize the two cases “fine-bedded” and “coarse-bedded” here because they show (as illustrated below) a clear discrimination in behavior. The transient, or “intermediated-bedded” cases show more ambiguous behavior.

A trend reflecting the effect of the density of bank/floodplain vegetation on bankfull Shields number seems to emerge in Fig. 1. In the coarse-bedded range ($D^{*}$ ≥  ~ 405, or $D_{50}$ ≥  ~ 16 mm), we find that the denser the bank vegetation, the higher is the bankfull Shields number (the blue points tend below the black trend line, and the purple ones tend more above it). This supports Parker et al.'s observation^42^ that the ratio of the bankfull Shields number to the critical Shields number increases with increasing bank vegetation density.

However, in the fine-bedded range ($D^{*}$ ≤  ~ 51, or $D_{50}$ ≤  ~ 2 mm) we do not see the same behavior. Instead, a perusal of Fig. 1 reveals that the denser the bank vegetation, the lower is the Shields number (the blue points tend to be above the black trend line, and the purple ones tend to be below it). This range includes all the locally identifiable sand-bed reaches and all the silt-bed reaches in the compendium used by Li et al.^37^. Thus there are three relevant ranges, the fine-bedded range, where denser bank vegetation correlates with lower Shields number, the coarse-bedded range where denser bank vegetation correlates with higher Shields number, and the intermediate-bedded range (2 mm to 16 mm) where the correlation is unclear.

**Figure 2:**
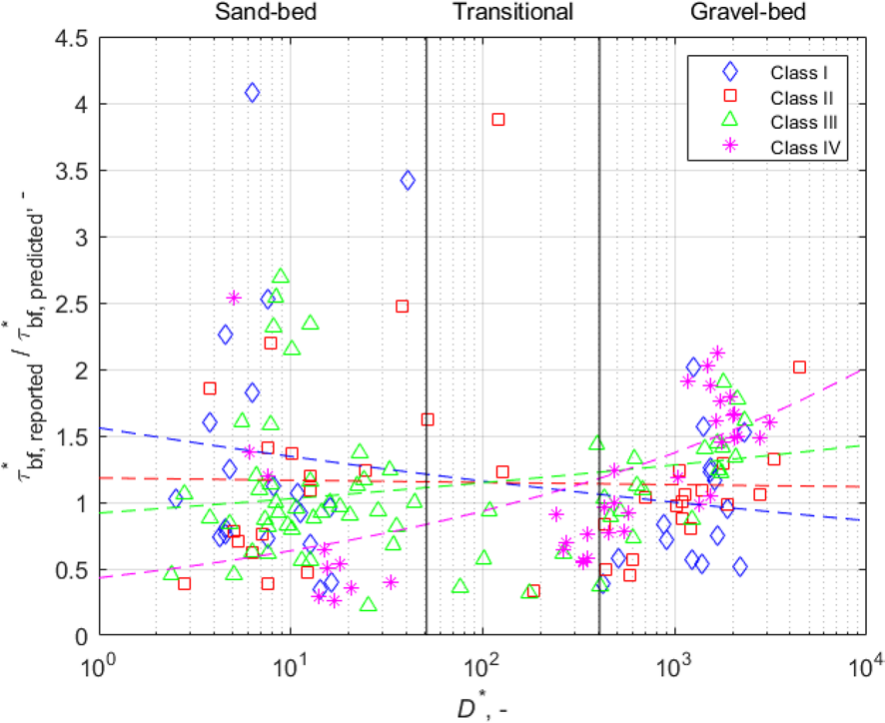
Fitted correction relations for the four woody vegetation cover density classes. The color scheme for the symbols is the same as Fig. 1.

These trends are more clearly shown in Fig. 2. It shows the ratio of each of the reported bankfull Shields numbers $\tau_{bf}^{*}$_,_ reported to the corresponding value $\tau_{bf\;predicted}^{*}$ predicted by Eq. (3), versus the corresponding dimensionless grain size D*. The data have been stratified according to vegetation density class. The mean values have been calculated for each bed material size range (fine-, intermediate- and coarse-bedded streams) in Table 1. The results in Fig. 2 and Table 1, show trends for the fine-bedded material range that are opposite to what we find for coarse-bedded material: as the vegetation density increases for $D^{*}$ ≤  ~ 51, the bankfull Shields number decreases monotonically. We show below that this result corresponds to channels that have wider bankfull widths as vegetation density increases.

**Table 1:** Average correction coefficients for each of four woody vegetation classes.

Tree and shrub cover^43^, *V*, -	Mean value of τ*_bf, reported_/τ*_bf, predicted_ (standard deviation)			Vegetation terms	
	D* < 51, or	*51* < D* < 405, or	405 < D*, or	*λ*_*A*_	*λ*_*B*_
	*D* < 2 mm	*2* < *D* < 16 mm	16 mm < *D*		
**Class I**					
0	1.392	–	0.953	1.559	− 0.0642
	(1.02)		(0.44)		
**Class II**					
0.01–0.05	1.199	1.862	1.009	1.18	− 0.0062
	(0.63)	(1.65)	(0.39)		
**Class III**					
0.05–0.5	1.087	0.829	1.182	0.92	0.0479
	(0.61)	(0.60)	(0.36)		
**Class IV**					
> 0.5	0.749	0.756	1.464	0.42	0.1704
	(0.58)	(0.21)	(0.44)		

Mean values also highlight the importance of the vegetation effect. In the range of fine-bedded material ($D^{*}$ < 51; $D_{50}$ < 2 mm), the bankfull Shields number is 39% higher than the average value prevailing for a floodplain without woody vegetation, while it is 25% lower in the case of the densest woody vegetation (Table 1). Figure 2 also shows power functions fitted to the points describing the relation between the ratio ($\tau_{bf\;reported}^{*} /\tau_{bf\;predicted}^{*}$) and $D^{*}$, again demonstrating that increasing bank vegetation density has the opposite effect on the bankfull Shields number in the fine-bedded cases ($D_{50}$ < 2 mm) than it does on the coarse-bedded cases ($D_{50}$ > 16 mm). The trend is not clear in the intermediate range, but this range includes only a small fraction (9.7% of the reaches used in Fig. 2).

In Table 1, the mean standard deviations for each grain size range are also shown in parentheses. These values suggest a stronger relationship between vegetation cover and Shields number for the coarse-bedded streams than for the fine-bedded streams.

In the following, we examine the relationships between bankfull Shields number $\tau_{bf}^{*}$, the two main riverbed geometric parameters ($B_{bf}$ and $H_{bf}$) and riparian woody vegetation root depth. The relationships have been examined separately for coarse-bedded and fine-bedded ($D^{*}$ > 405 and D* <  ~ 51) reaches, and for cases where woody vegetation is observed (Class II, Class III, and Class IV, following the Hey and Thorne classification^43^). In the following analysis, we consider the bankfull depth-independent data set of discrepancy ratios of $\tau_{bf\;reported}^{*} /\tau_{bf\;predicted}^{*}$ values described in Methods, so as to focus on the role of root depth.

**Figure 3:**
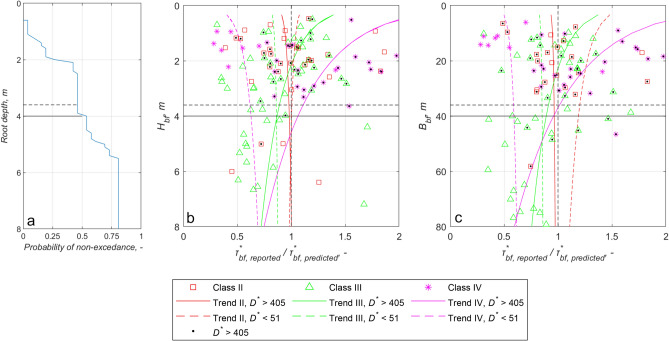
(**a**) Probability of non-exceedance of root deth as a function of depth below surface. (**b**,**c**) Trend analysis of correction ratios τ _bf_*, _reported_/τ _bf_*, _predicted_ as functions of bankfull depth (H_bf_) (**b**) and bankfull width (B_bf_) (**c**), respectively. Continuous horizontal black line shows the most probable root layer ($H_{root\_median} = 4.0\,{\rm m}$) in (**a**,**b**) and $10H_{root\_median}$ in (**c**). Dashed horizontal black line shows the mean root layer ($H_{root\_mean} = 3.6\,{\rm m}$) in (**a**,**b**) and $10H_{root\_mean}$ in (**c**).The data are discriminated according to vegetation class and bed material. The colors and symbols refer to the riparian vegetation density: red square—Class II, green triangle—Class III and pink star—Class IV (the densest vegetation cover). Granular ($D^{*}$ >  ~ 405) bank material are denoted by a black dot in the symbol, while the symbols for finer soils ($D^{*}$ <  ~ 51; D <  ~ 2 mm) are unfilled. The coloring order of the fitted curves is the same as that of the symbols. The solid lines represents the granular ($D^{*}$ >  ~ 405; D >  ~ 16 mm) sediment, while the dashed lines represent the finer sediment ($D^{*}$ <  ~ 51). We did not display Class 1 in the figure because there was no depth dependent trend (which is to be expected due to the lack of vegetation).

It is not possible for us to measure rooting depths for all 175 reaches. We thus used the data subset of Stromberg^32^ consisting of root depth values of solely riparian woody vegetation as a benchmark for statistical purposes. To this end, Fig. 3a shows the cumulative distribution of root depth. The realistic depth of the root zone, as well as the most probable (median) value can be inferred from this figure ($H_{root\_median} = 4.0\;{\text{m}}$, indicated by a continuous horizontal line in each of Fig. 3a,b). Figure 3b,c show the trend-free discrepancy ratios $\tau_{bf\;reported}^{*} /\tau_{bf\;predicted}^{*}$, where the $\tau_{bf\;predicted}^{*}$ values were calculated according to Li et al.^36^ (Eq. 3). In the same Figures, the horizontal axis shows the bankfull depth (Fig. 3b) and width (Fig. 3c). The solid colored lines pertain to rivers with the coarse-bedded material ($D^{*}$ >  ~ 405), and the dashed lines pertain to those with fine-bedded material ($D^{*}$ <  ~ 51). Points in the transitional range (51 < $D^{*}$ <  ~ 405) were omitted from these figures.

The fitted curves in Fig. 3b show that as long as the bankfull depth is less than a value near median root depth, the bankfull Shields number (a) increases as the vegetation density intensifies in the case of coarse-bedded material ($D^{*}$ >  ~ 405, solid lines), but on the other hand (b) decreases for finer-bedded material ($D^{*}$ <  ~ 51, dashed lines). These trends are no longer present if the bankfull channel is deeper than the value around the median of the root depths. The curves also show that the effect of vegetation is greatest when the ratio $H_{bf} /H_{root\_median}$ value is the smallest. As this ratio increases—i.e., the larger the thickness of the potential rootless zone in the riverbank—vegetation density classes separate the points less and less, and the role of the root zone gradually decreases. Laboratory investigation of the root reinforcement of the botanical species Picea Abies delivered the same outcome^13^. That is, the root reinforcement decreases nonlinearly with increasing vertical distance below tha bank surface, and at distances larger than the typical root length (2.5 m in the case of Picea Abies), no considerable root reinforcement can be observed^13^. Moreover, the left of Fig. 3b also suggests that the dependence on bed material (i.e. bottom bank material) is no longer present below the most likely (median) root depth: the dashed and solid lines take on the same order values.

On the right side of Fig. 3c, we can see from the logarithmic curves fitted to the points that in the range of relatively small bed width ($B_{bf}$ < $10H_{root\_median}$), the previously established trends (Fig. 2 and Table 1) apply to river sections with different woody vegetation densities (Class II, III, and IV, following Hey and Thorne calssification^43^) and bank material ($D^{*}$ < 51 and > 405). It can also be seen that the narrower the channel (i.e. $B_{bf}$ < $10H_{root\_median}$), the more the different points are separated from each other, indicating the increasing role of vegetation. However, as bed width increases, the curves approach each other, and the vegetation-dependent behavior postulated in the hypothesis disappears.

## Increasing floodplain woody vegetation density renders coarse-bedded rivers narrower but fine-bedded rivers wider

The ranges “fine-bedded” (*D* ≤ 2 mm) and “coarse-bedded” (*D* ≥ 16 mm) loosely correspond to sand-bed and gravel-bed streams. (Technically speaking, gravel is often defined as *D* > 2 mm, and there are a few silt-bedded streams in the collection used here.)

One of the most accurate relations for total bed material load of sand-bed streams is that of Engelund and Hansen^48^.This is illustrated in, for example, Ma et al.^49^. When applied to bankfull flows, the relation takes the form

4 $$Q_{tbf} = \frac{0.05}{{C_{f} }}\sqrt {RgD} DB_{bf} \left( {\tau_{bf}^{*} } \right)^{5/2} ,$$

where bed friction coefficient $C_{f}$ can be estimated from the normal flow assumption as

5 $$C_{f} = \frac{{gH_{bf} S}}{{U_{bf}^{2} }} ,\quad U_{bf} = \frac{{Q_{bf} }}{{B_{bf} H_{bf} }}.$$

Here *U*_*bf*_ denotes cross-sectionally-averaged flow velocity at bankfull flow.

Now we assume that *Q*_*tbf*_, *R*, *D* and and *C*_*f*_ are held constant. From Eq. (4), then, higher values of *τ*_bf_* are specifically associated with lower values of *B*_*bf*_. We have shown above that in fine-bedded streams, increasing density of riparian vegetation is associated with a reduction in bankfull Shields stress *τ*_bf_*. That is, we obtain the somewhat counterintuitive result that increased density of woody vegetation correlates with wider, rather than narrower channels in fine-bedded streams.

A similar argument can be used for coarse-bedded streams. For these, we assume that gravel moves as bedload, and sand moves as wash load over the gravel bed. Thus the volume bedload transport rate at bankfull flow can be approximately equated with the volume total bed material load *Q*_*tbf*_.

For the sake of illustration, we adopt the bedload transport rate of Parker^50^, which represents an approximation of the Einstein^51^ bedload transport relation, but in explicit form. At bankfull flow, it takes the form

6 $$Q_{tbf} = 11.2\sqrt {RgD} DB_{bf} \left( {\tau_{bf}^{*} } \right)^{1.5} \left( {1 - \frac{{\tau_{c}^{*} }}{{\tau_{bf}^{*} }}} \right)^{4.5} ,$$

where *τ*_c_^*^ = 0.03 denotes a critical Shields number for the onset of motion. Again, if *Q*_*tbf*_, *R*, *D* and *τ*_c_^*^ are held constant, increasing *τ*_bf_^*^ is associated with a narrowing channel, i.e. the same tendency as shown by the sand-bed relation of Engelund and Hansen^48^. We have shown above, however, that in the case of coarse-bedded rivers, increasing density of woody vegetation causes an increase in *τ*_bf_*. Thus according to Eq. (6) and the trends in Fig. 2, increasing woody vegetation density should result in a narrower bankfull channel. The same tendency is obtained using, for example, the gravel transport relation of Meyer-Peter and Muller^52^. This result concerning coarse-bedded streams is in agreement with analysis of Hey and Thorne^43^ for gravel-bed streams.

For emphasis, we summarize the essential conclusion of this paper: (a) in coarse-bedded streams, increasing density of woody vegetation is associated with an increase in bankfull Shields stress, and thus a decrease in bankfull width; whereas (b) in fine-bedded streams, increasing density of woody vegetation is associated with a decrease in bankfull Shields stress, and thus an increase in bankfull width.

## Discussion

With the help of the functions fitted to the set of points separately for each class, we introduce an amended version of the Li et al.^36^ equation for estimating the bankfull Shields number. This way, the effect of vegetation on the alluvial river bankfull geometry can be taken into account when estimating the bankfull Shields number. The correction, optimized using the method by Li et al.^36^ takes the form:

7 $$\tau_{bf}^{*} = \lambda_{A} 502D^{{{*}\left( {\lambda_{B} - 0.951} \right)}} S^{0.434} ,$$

where $\lambda_{A}$ and $\lambda_{B}$ are parameters dependent on woody vegetation density. The values were determined according to the best fit principle for each vegetation cover class (see the last two columns of Table 1). The validation of the derived relations is detailed in the “Methods” section. Here, instead of discrete classes, we characterize the bank vegetation cover in terms of a continuous variable useful in numerical modeling. For this purpose, the dimensionless parameter *V* estimates the areal bank/floodplain vegetation cover fraction, so as to extend the classification of Hey and Thorne^43^*.* (*V* can take any value between 0 and 1, where 0 means tree- and shrub-free bank and 1 represents full tree-shrub coverage. The method of determining areal bank vegetation cover fraction is detailed in the “Methods” section. The amended bankfull Shields number can be calculated for any bank vegetation cover fraction (*V*) using the following regression relationships in Eq. (7):

8 $$\lambda_{A} = 1.424e^{{ - 1.562{ }V}} ,\;{\text{and}}$$

9 $$\lambda_{B} = 0.0003{ }V^{2} + 0.2768{ }V - 0.0413.$$

These relationships apply to the complete range of grain sizes studied here, including fine-bedded, intermediate-bedded and coarse-bedded.

The average of the discrepancy ratios $\tau_{bf\;reported}^{*} /\tau_{bf\;predicted}^{*}$, using the original form (Eq. 3) and the amended form (Eq. 7, where $\lambda_{A}$ and $\lambda_{B}$ are calculated by Eqs. 7, 8 and 9), respectively, are as follows for the four classes: Class I, 1.20 and 0.99; Class II, 1.18 and 1.03; Class III, 1.11 and 1.06; Class IV, 1.16 and 0.98. The ratios suggest that the Li et al.^36^ prediction works most accurately in Class III; that is, it was (inadvertently) optimized for average vegetation cover fraction in the range 0.05 < *V* < 0.5. Therefore, if the effect of woody vegetation is ignored, the smallest error is expected for Class III. Otherwise, the correction presented here can still significantly improve the prediction of bankfull Shields number, i.e., up to 19%.

**Figure 4:**
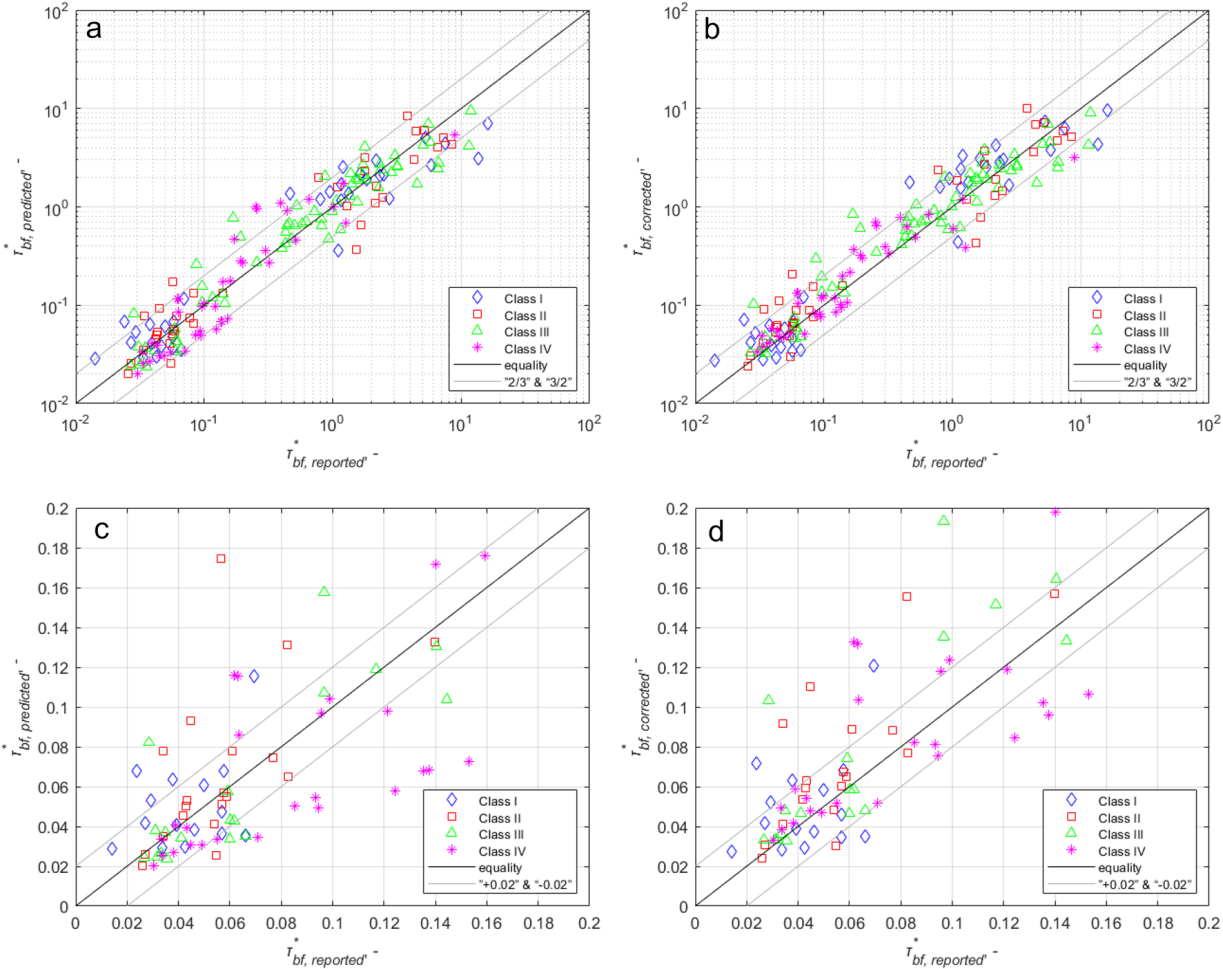
Panels (**a**,**c**) show points predicted by Li et al.’s estimation versus the reported bankfull Shields number $\tau_{bf}^{*}$. (**b**,**d**) show predictions from Eq. (2) versus reported bankfull Shields number $\tau_{bf}^{*}$. The colors and symbols refer to the riparian vegetation density, as shown in the legend. The two figures on the left show the prediction by Li et al., and the figures on the right show the results of the improved relationship (Eq. 2). The lower two figures (c and d) show the range 0–0.2 of the vertical axis. This range more clearly illustrates the improvement. The solid black line shows equality, and the gray lines the 20% margin of error.

The effect of the correction is illustrated in Fig. 4. The horizontal axes show the reported Shields numbers. The vertical axes of the left figures show the Shields numbers corrected by Eq. (7) and the vertical axes of the right figures show the Shields numbers estimated by the original Li et al.^36^ relation. In the upper two figures, the range of axes is 0.01 to 100, while in the lower figures, they are 0 to 0.2. [The figures show that a noticeable improvement is seen mainly in the range below 0.2, i.e., for coarser material (D* >  ~ 51). This is consistent with the standard deviation discrepancy ratio values for coarse and fine material in Table 1: since the average error can be described typically with a smaller standard deviation in the case of coarser material (D* >  ~ 51), thus a better improvement with the correction equation is also expected in this range.

It can be seen in Fig. 2 that the expected effect of riparian vegetation in the case of sand-bed rivers is observed clearly for a range of about D* < 100, since the trend lines intersect around 100. Similarly, the expected behavior observed for gravel-bed rivers is observed clearly for D* > 600. The range 100 < D* < 600 (4 mm < *D* < 24 mm) appears as a transition where the effect of riparian vegetation is not clear. This range shows a substantial overlap with a transitional range of 51 < D* < 405 (2 mm < *D* < 16 mm) according to the classification established by Parker^44^. The transitional range obtained according to our study and determined by Parker thus at least loosely correspond: the sand- and gravel-bed rivers can be separated from each other, and there is a transition between the two (corresponding to a range where reaches are rather rare) in which the bed material is neither sand- nor gravel dominated, and thus the role of riparian vegetation is also unclear in terms of impact on bankfull Shields number.

**Figure 5:**
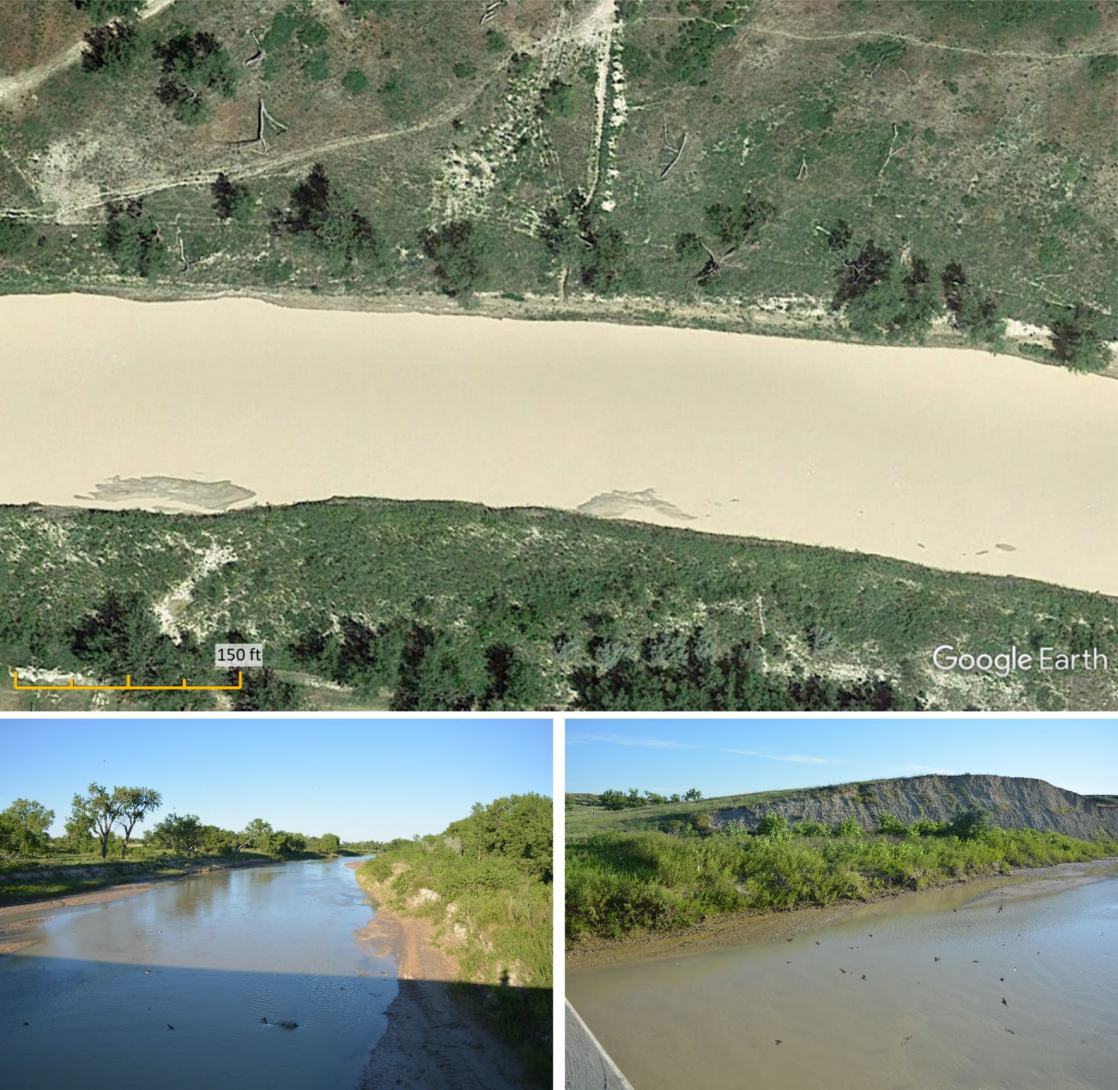
White River at Interior, South Dakota, USA (top image: Maps Data: Google, © 2022, lower images: own photos).

The following is a simple example of the applicability of the riparian vegetation-based relationships (Eqs. 7, 8 and 9) introduced in this paper. The Wilkerson et al.^41^ data set includes a river section of the White River at Interior, South Dakota, which has a bed grain size *D* = 0.5 mm (Fig. 5). Based on the on-site tour of the authors (and also photos in Fig. 5), the river section can be classified as Class 2 (1–5% vegetation cover) following the classification of Hey and Thorne^43^. This class yields the estimates $\lambda_{A} =$ 1.37 and $\lambda_{B} = { } - 0.034$ from Eqs. 8 and 9. Taking into account the reported slope ($S = 0.002$) and grain size ($D = 0.5$ mm) values, the bankfull Shields number is obtained as $\tau_{bf}^{*} = 3.802$ (Eq. 7), from which (applying the Engelund and Hansen formula, Eq. 4 and 5) $B = 121$ m can be estimated. The reported values^41^ are $\tau_{bf}^{*} = 4.286$ and $B = 89$ m, while the values estimated by the original relation of Li et al.^36^ (combined with Engelund and Hansen) are $\tau_{bf}^{*} = 3.029$ and $B = 213$ m. With the help of the correction equation (Eq. 7), the section width belonging to different degrees of riparian bank cover can be estimated: $B = 114$ m (decrease to 94% of the width calculated for the current woody vegetation cover - Class 2) in the case of very sparse woody bank vegetation (*V* = 0), while in the case of *V* = 0.3 (30% woody vegetation cover), the width $B = 213$ m estimated by the relation of Li et al.^36^ would be obtained (corresponding to a 76% increase compared to the current woody vegetation cover - Class 2).

As noted above, the regression relation of Li et al.^37^ has been used as the basis for the analysis. Czapiga et al.^38^ offer a similar relation. The use of this latter relation yields similar results that do not change our essential conclusions.

## Summary and conclusions

In this study we investigate how riparian woody vegetation affects the morphodynamic characteristics of rivers. Our key results can be stated as follows.

1. In a fine-bedded range (*D* ≤ 2 mm), increasing woody vegetation density tends to cause a decrease in bankfull Shields number, and a corresponding increase in bankfull width.
2. In a coarse-bedded range (*D* ≥ 16 mm) increasing woody vegetation tends to cause an increase in bankfull Shields number and a corresponding decrease in bankfull width.
3. The behavior is mixed in an intermediate range (2 mm < *D* < 16 mm).

We provide a set of dimensionless, empirical relations with which to estimate the effect of increasing woody density on bankfull Shields number across the entire range of grain sizes studied here (0.094 mm ≤ *D* ≤ 175.8 mm).

The effect of vegetation on equilibrium bankfull geometry can be significant. It affects not only the bed width but also the water depth. According to the calculation for the White River at Interior presented above, it can cause an increase or decrease on the order of 10% of bankfull width and depth, but in extreme cases, it can even result in an order of magnitude change. Such variation is also pertinent to the prediction of flood water levels, as well as the management of temperature in rivers. The analysis presented here has a potentially useful application as regards climate change. Climate change is predicted to have major effects on vegetation regime^53^. Specifically, possible effects of climate change might be a decrease in riparian hardwood species or density, and the spread of more drought tolerant shrubs^54^. Our analysis can help predict how changing bank and floodplain vegetation can affect river channel geometry.

Our findings merit consideration by river managers. The result concerning coarse-bedded rivers, i.e. that increasing vegetation density is correlated with a narrower channel, is straightforward. The result concerning fine-bedded rivers, i.e. that increasing vegetation density is correlated with a wider channel, is counterintuitive, yet supported by our data analysis. We suggest that river managers take these trends into account, while balancing them against other factors such as ecosystems management, flood control, recreational use etc.

## Methods

The data used in this study were generated, as a first step, by filtering the data employed by Wilkerson et al.^41^. From that set we selected only the river sections whose exact location we were able to determine. Thus, 85 river sections were assigned to a fine-bedded range (bed material median size *D*_50_ ≤  ~ 2 mm), 56 were assigned to a coarse-bedded range (bed material median size *D*_50_ ≥  ~ 16 mm) and 16 more were assigned to an intermediate-bedded range (2–16 mm). The rest of the data used here are 61 river sections from the database in the publication by Hey and Thorne^43^; i.e., the database referred to in Parker et al.^42^ publication as the Britain II data set. All data used in this analysis are part of the Li et al.^37^ data set, and are available in the referenced publications.

**Figure 6:**
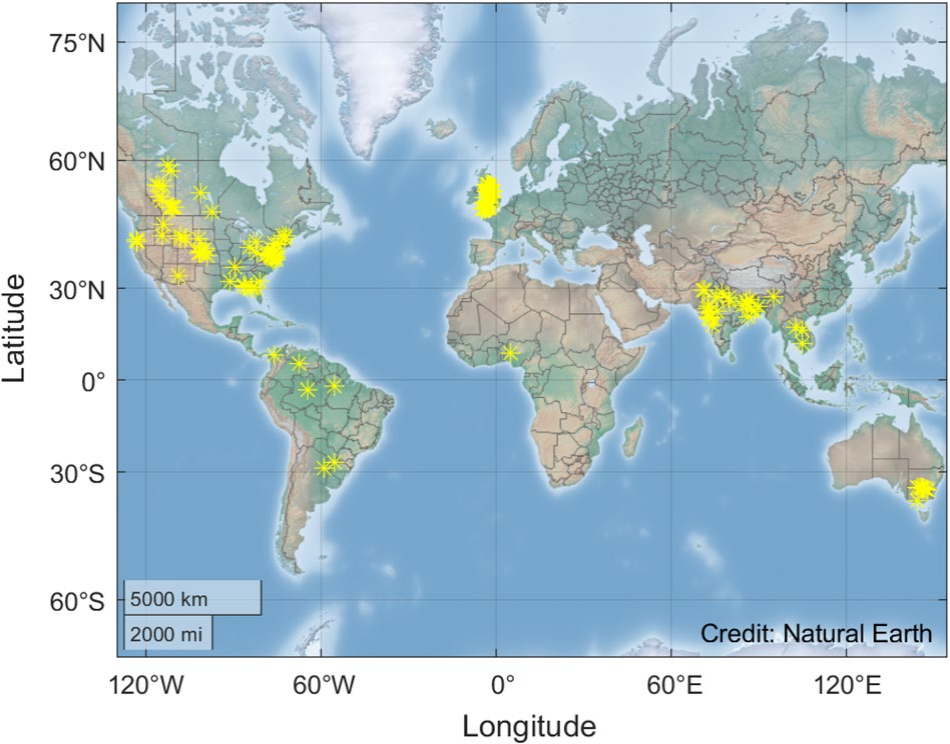
Locations of the investigated river sections.

As opposed to bedrock rivers, vegetation is expected to play a significant role in the morphodynamic processes of alluvial rivers^34^. Therefore, we investigated only alluvial channels in order to quantify the role of riparian vegetation. The data set includes both temperate and tropical streams, as shown in Fig. 6. All the streams are between ± 66.5° latitude. No streams in permafrost are included in our set. In such streams, the effect of ice likely plays a more important role in channel morphology than vegetation, the root depth of which may be severely restricted by permafrost^35^. We have thus chosen reaches such that the effects of polar climate and bedrock can be neglected, and so the morphodynamic processes are primarily determined by the interaction of water, sediment and vegetation^55^. In this regard, we follow the lead of several studies^37,38,41,42^.

Typical plant species may be different in different river sections, in particular if they are located in different climate regimes. In this study, there was no universal and straightforward way to identify the vegetation type at each site via remote sensing, and visiting all river sections would have been an unrealistic undertaking. Although different tree species have different physiological properties^56^, they show the same behavior in that the denser the plant, the higher the load for the bank and the more branched the root system in the channel bank. The basis for our study of the effect of riparian woody vegetation on river morphodynamics is an analysis of our data set.

The vegetation coverage for each river section presented in the data set of Wilkerson et al.^41^ and Parker et al.^42^ was determined using Google Earth. The examined sections are marked by yellow stars in Fig. 6.

**Figure 7:**
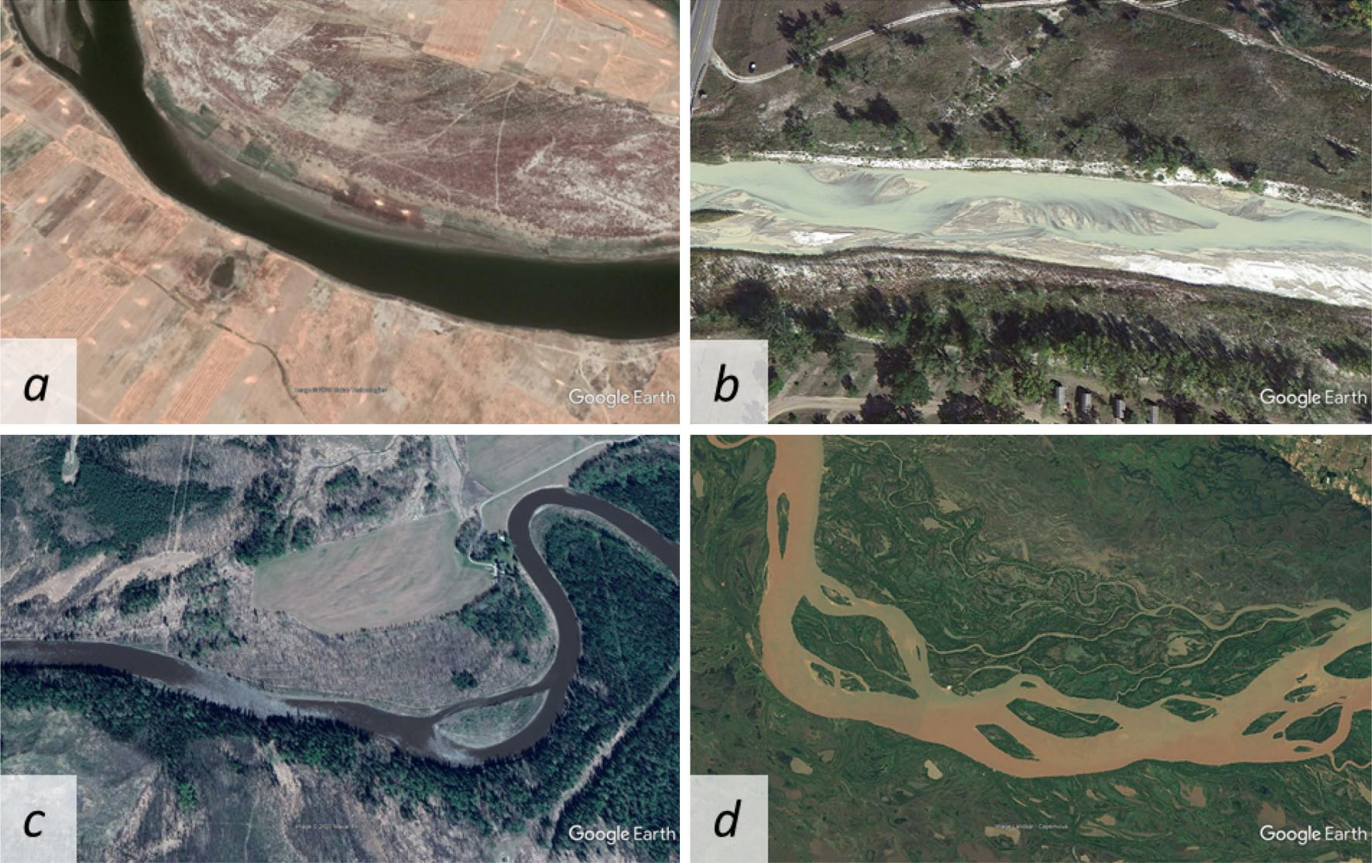
(**a**) Ramganga River, in Uttar Pradesh state, India—Class I (Maps Data: Google, © 2022 Maxar Technologies) (**b**) White River at Interior, South Dakota, USA—Class II (Maps Data: Google, © 2022) (**c**) Pembina River near Paddy Creek, Alberta, Canada—Class III (Maps Data: Google, © 2022 Maxar Technologies) (**d**) Rio Parana, Argentina—Class IV (Maps Data: Google, © 2022 Image Landsat/Copernicus).

The classification of Hey and Thorne^43^ for vegetation cover was determined by visual inspection. To determine the vegetation cover, a fairly long bank section of a few hundred meters at any given river section was examined, and the vegetation along banks was considered with equal weight. The first class includes those river sections on the banks of which neither trees nor bushes are visible at all (Fig. 7a). In the second class, similarly to the first, the undergrowth may be more widespread, but rarely and erratically, with as little as a single tree appearing on the bankline in the image we used. (Figs. 5, 7b). In the third group, the trees are more dense, forming a forest; but for the two banklines of the entire studied section, they may cover a maximum of half of the bankline (Fig. 7c). Vegetation with a density and bankline in excess of the above was classified as Class 4 (Fig. 7d). An example of each member of the four classes is shown in Fig. 7.

Characteristic bankfull geometry can be examined based on a consistent equation system that can be constructed from the following three equations:

- Water mass balance equation:
  10 $$Q_{bf} = U_{bf} B_{bf} H_{bf} ,{\text{where}}$$
  $Q_{bf}$ is bankfull water discharge [m^3^/s], $U_{bf}$ is the bankfull flow velocity [m/s], $B_{bf}$ [m] and $H_{bf}$ [m] are the bankfull width and depth, respectively.
- Normal flow momentum equation:
  11 $$\left[ {\frac{{\tau_{bf} }}{\rho } = } \right]C_{f} U_{bf}^{2} = gH_{bf} S,{\text{where}}$$
  $\tau_{bf} \left[ { = \tau_{bf}^{*} \left( {\rho_{S} - \rho } \right)gD} \right]$ is the bankfull bed shear stress [N/m^2^], $\rho_{S}$ is the density of sediment (2650 kg/m^3^), $\rho$ is water density (1000 kg/m^3^), $C_{f}$ is the dimensionless bed friction coefficient, *g* is gravitational acceleration (9,81 m/s^2^) and *S* is slope [-].
- Sediment mass balance equation (see Eqs. 4 and 6).

Using the behavior of the data in Fig. 2, we attempt to improve the Li et al.^36^ equation for estimating the bankfull Shields number. Our investigation is based on the calculation of discrepancy ratios $\tau_{bf\;reported}^{*} /\tau_{bf\;predicted}^{*}$: we sorted them into four classes according to vegetation density. We then fitted a curve to each of the four classes (Fig. 2). The values of $\lambda_{A}$ and $\lambda_{B}$ (woody vegetation density-dependent parameters, Eqs. 7, 8 and 9) were obtained accordingly (Table 1).

The correlations we extracted from the data were verified as follows: the values of discrepancy ratios were randomly split in a ratio of a) one-third to b) two-thirds, separately for all four vegetation classes. Then, power functions were fitted to the data for group b) (two-thirds), based on the values of $\lambda_{A}$ and $\lambda_{B}$ calculated with that group. Finally, the accuracy of the fitted curve was tested using data group a), here considered as data independent from group b). This process was repeated 1000 times: the cumulative accuracy ratios ($\tau_{bf\;reported}^{*} /\tau_{bf\;predicted}^{*}$) (i.e. within a given class, the average of the accuracy ratios calculated up to a given number of cycles) were also calculated after each cycle.

**Figure 8:**
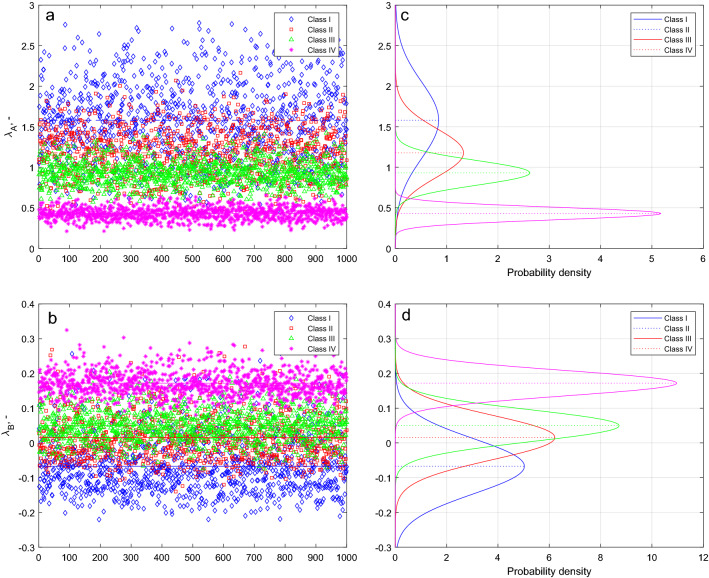
The values of parameters $\lambda_{A}$ (**a**, upper left) and $\lambda_{B}$ (**b**, lower left) are calculated from the power curves fitted to the randomly selected points. Random selection and determination of parameters were performed 1000 times. Figures 8c and 8d on the right respectively show the normal distribution fitted to the $\lambda$ values.

Figures 8a (upper) and 8b (lower) show the calculated values of $\lambda_{A}$ and $\lambda_{B}$ for each cycle (a total of 1000 calculation cycles, as shown in the x axes). According to the results of the t-test performed separately for a total of 8 sets of points, the null hypothesis is rejected at the 5% significance level. In other words, since there is less than a 5% probability that the value obtained for the λ values is the result of chance, the function fitting can be considered statistically significant (Fig. 8c,d).

It can be seen in Fig. 8 that although the scatter of the points belonging to each class is significant, the point clouds of different classes typically fall into different ranges. The symbols in Fig. 8a show at which value the fitted correction curves cross the y-axis in Fig. 2. Based on this, the relative positions of the differently-colored clouds confirms the earlier finding: in the case of fine-bedded material, the denser the bankfull vegetation, the smaller the Shields number and the wider the channel. Figure 8b represents the exponent of the correction power functions. Despite the significant variance, there is a clear trend in the relative position of point clouds of different colors. Based on interpretation of the exponents (see Fig. 2 and Eq. 2), it is found that in coarse-bedded material, vegetation increasingly raises the Shields number and narrows the channel. The average values of the point clouds are displayed in Table 1 ($\lambda_{A}$ and $\lambda_{B}$), and the correction curves fitted to them are shown in Fig. 2.

These findings show that that the expected error of the relation by Li et al.^37^ can be characterized in terms of riparian vegetation density. Accordingly, correction can be made using Eqs. (8) and (9).

The values for cumulative accuracy also serve to validate our relations for correction, Eqs. (8) and (9), based on woody vegetation cover. The following accuracy ratios ($\tau_{bf\;reported}^{*} /\tau_{bf\;predicted}^{*}$) were obtained for an arbitrary computation: After 10 cycles: Class 1: 1.12; Class 2: 0.86; Class 3: 1.09; Class 4: 1.17. After 100 cycles: 1.02; 0.99; 1.04; 0.98. After 1000 cycles: 1.02; 1.01; 1.00; 1.01. Based on these numbers, it can be seen that on the one hand, there is a significant scatter and a perfect match is not expected. On the other hand, the significant trend in cumulative accuracies toward unity confirms that the density of riparian vegetation does indeed affect the Shields number, according to our hypothesis.

**Figure 9:**
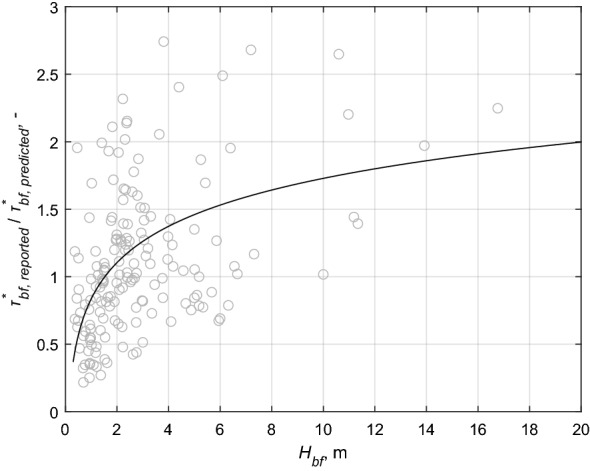
Determination of the water depth-dependent trend $T\left( {H_{bf} } \right)$ in the $\tau_{bf\;reported}^{*} /\tau_{bf\;predicted}^{*}$ data set. The solid line shows the prediction of $\tau_{bf\;reported}^{*} /\tau_{bf\;predicted}^{*}$ as the function of $H_{bf}$. See Eq. (12) below.

We investigated the dependency of the discrepancy ratios of $\tau_{bf\;reported}^{*} /\tau_{bf\;predicted}^{*}$ on bankfull depth $H_{bf}$, so as to determine whether the error of the estimate proposed by Li et al.^37^ can be predicted as a function of $H_{bf}$. Figure 9 shows the relationship between these values.

Using all of our points, we found a logarithmic trend for the discrepancy ratio $\left( {\tau_{bf}^{*} } \right)_{reported} /\left( {\tau_{bf}^{*} } \right)_{predicted}$ as a function of bankfull depth $H_{bf}$ for all the points. The value of each point ($Y$) can be partitioned into a bankfull water depth-dependent trend ($T$) and a scatter ($S$) term, in accordance with the following form:

12 $$Y = T\left( {H_{bf} } \right) + S.$$

According to the equation, each value ($Y$) can be written as the sum of $T\left( {H_{bf} } \right)$ and an irregular term, representing the water depth-independent term ($S$).

The trend we detected in Fig. 9 can be described by the following equation:

13 $$T\left( {H_{bf} } \right) = 0.3876\ln H_{bf} + 0.8358.$$

This trend suggests that the accuracy of the bankfull Shields number prediction introduced by Li et al.^37^ depends on bankfull depth. On the other hand, the coefficient of determination ($R^{2}$) of 0.39 also shows that additional factors likely play roles in the establishment of the bankfull Shields number. To detect them (in our specific case: the impact of riparian woody vegetation), we filter out the dependence of the discrepancy ratios on the bankfull depth. Thus, the bankfull depth-independent data set can be calculated as follows:

14 $$S = Y - T\left( {H_{bf} } \right) = Y - 0.3876\ln H_{bf} - 0.8358.$$

## Data Availability

All data used in this examination are available in Wilkerson et al.^41^ and Parker et al.^42^.

## List of symbols

$$B_{bf}$$: Bankfull width
$$H_{bf}$$: Bankfull depth
$Q_{bf}$: Bankfull water discharge
$U_{bf}$: Cross-sectional-averaged bankfull flow velocity
$$S$$: Bed slope
$D_{50}$: Median bed material grain size
$R$: Submerged specific gravity of bed material
ν: Water kinematic viscosity
*g*: Gravitational acceleration
$\tau_{bf}^{*}$: Bankfull channel Shields number
$\tau_{bf}$: Bankfull bed shear stress
$$\rho_{S}$$: Sediment density
$$\rho$$: Water density
$\tau_{c}^{*}$: Critical Shields number for the onset of particle motion
$D^{*}$: Dimensionless bed material grain size
$C_{f}$: Bed friction coefficient
$\lambda_{A}$ and $\lambda_{B}$: Parameters dependent on woody vegetation cover
*V*: Frontal bank/floodplain woody vegetation cover fraction
